# An elevated 8-isoprostaglandin F2 alpha (8-iso-PGF2α) in COVID-19 subjects co-infected with malaria

**DOI:** 10.11604/pamj.2020.37.78.25100

**Published:** 2020-09-21

**Authors:** Yahaya Muhammad, Yamuna Kani Aminu, Abdurrahman Elfulaty Ahmad, Sani Iliya, Nuruddeen Muhd, Mohammed Yahaya, Aminu Sale Mustapha, Abdulkhabir Tahiru, Sulaiman Saeed Abdulkadir, Jamila Suleiman Ibrahim, Abdulmalik Binji Ahmad, Idris Yahaya Muhammad, Zaharaddeen Shehu, Abdulrahman Yakubu, Bashir Kabir Muhd, Armaya’u Ahmed, Umar Abubakar Faruk

**Affiliations:** 1 Department of Chemical Pathology Rasheed Shekoni Teaching Hospital Dutse, Jigawa, Nigeria,; 2 College of Medicine and Health Sciences, Federal University Dutse, Jigawa, Nigeria,; 3 Department of Medical Laboratory Science, Ahmadu Bello University Zaria, Nigeria,; 4 Department of Biological Sciences, School of Pure and Applied Sciences, Mount Kenya University Thika, Thika, Kenya,; 5 Medical Laboratory Department, General Hospital Dutse, Kiyawa Road, Jigawa, Nigeria,; 6 Department of Hematology, Rasheed Shekoni Teaching Hospital Dutse, Jigawa, Nigeria,; 7 Department of Pathology 44 Nigerian Army Reference Hospital, 7 Sokoto Road Kaduna, Kaduna, Nigeria,; 8 State Ministry of Health Kaduna, Kaduna, Nigeria,; 9 Federal Neuropsychiatric Hospital, Pathology Department, Kware-Sokoto, Nigeria,; 10 Department of Science Laboratory Technology, Jigawa State Polytechnic, Jigawa, Nigeria,; 11 Department of Biotechnology Federal University Dutse, Jigawa, Nigeria,; 12 Department of Chemical pathology Usmanu Danfodiyo University Teaching Hospital Sokoto, Nigeria,; 13 Public Health and Diagnostic Institute, Northwest University Kano, Nigeria

**Keywords:** COVID-19, malaria, oxidative stress

## Abstract

**Introduction:**

the most recently discovered severe acute respiratory syndrome Coronavirus 2 (SARS-COV-2) that causes COVID-19, subjected the entire world in turmoil health-wise and economically. With higher burden of malaria in Nigeria and other sub-Saharan African countries coupled with fragile healthcare system and delivery, these may pose a threat in the diagnosis and management of COVID-19 patients co-infected with malaria. Free radicals have been implicated in the progression and pathogenesis of malaria and COVID-19 through Fenton’s reaction and cytokine storm respectively.

**Methods:**

the current research comprises of seventy-four (74) participants; 20 apparently healthy controls and 54 COVID-19 patients (34 among which were co-infected with malaria). Serum levels of 8-iso PGF2α and Alphatocopherol were determined among the study participants using ELISA technique and colorimetric assay, respectively.

**Results:**

results revealed statistically significant elevation of 8-iso PGF2α in COVID-19 patients co-infected with malaria compared to COVID-19 patients only, and this may be due to increase production of free radicals. Furthermore, a significant decrease of Alphatocopherol was observed in COVID-19 co-infected with malaria compared to COVID-19 patients due to increase utilization of antioxidants in counterbalancing the negative effect of free radicals generated.

**Conclusion:**

conclusively, SARS-COV-2 patients co-infected with malaria might be predisposed to oxidative stress and low Alphatocopherol. The increase in oxidative stress is proportional to malaria parasite density and inversely related to Alphatocopherol levels. This implies that oxidative stress is notably higher and such patients may have a severer form of the COVID-19. Increased 8-iso-PGF2α in co-infection and decreased alphatocopherol levels can reflect the severity and adverse outcomes compared to COVID-19 naïve because of their tremendous involvement in the pathogenesis and progression of diseases.

## Introduction

In early December 2019, several cases of pneumonia were reported in Wuhan city, Hubei province of China. The causative agent was later identified as severe acute respiratory syndrome coronavirus 2 (SARS-COV-2) and the disease named as COVID-19. SARS-COV-2 attaches to ACE 2 receptors by spike glycoprotein in epithelial cells of gastrointestinal tract, nose, pharynx where it replicates and travels to other organs [1]. In the lungs, interaction of different immune active molecules such as interleukins, chemokines, colony-stimulating factor and tumor necrotic factor alpha (TNF-α) ensues thereby triggering proinflammatory response ultimately leading to respiratory distress syndrome (RDS), septic shock and multiple organ failure in severe cases with consequent formation of free radicals [2]. Since the COVID-19 pandemic first emerged, the disease has affected more than 200 countries and territories with over 10 million cases and about 502,000 deaths globally [3]. Africa is the least continent affected with approximately 100,000 reported cases and more than 2,500 deaths (as at 28 June, 2020) [4]. With higher burden of malaria in Nigeria and other sub-Saharan African countries coupled with fragile healthcare system and delivery, these may pose a major threat in the diagnosis and management of COVID-19 patients co-infected with malaria [5]. Malaria is a mosquito-borne parasitic infection spread by female Anopheles mosquitoes via a single-celled parasite known as plasmodium that multiplies in human erythrocytes as well as mosquito intestine [4-6]. Oxidative stress has been implicated in the pathogenesis and progression of both malaria and COVID-19 via Fenton´s reaction and cytokine storms respectively [7,8]. Oxidative stress may be defined as overproduction of free radicals overwhelming the antioxidants defense system leading to destruction of lipid membranes via chain reaction with formation of by-products such as malondialdehyde (MDA), 8-iso-PGF2α, lipoperoxides, lipid hydroperoxides and hydroxy nonenal alkyne [9]. Sole determination of free radicals is difficult due to their unstable and short half-life nature, instead their metabolites [9]. 8-iso-PGF2α is considered as the most stable and reliable biomarker of oxidative stress (gold standard) attribute to its sensitivity and specificity [10]. Current study aimed at determining the serum levels of 8-iso PGF2α and alphatocopherol in COVID-19 patients co-infected with malaria and association between 8-iso-PGF2α and Alphatocopherol COVID-19 naïve and co-infected.

## Methods

**Study population:** the study consisted of 74 participants, comprising of 54 patients diagnosed with COVID-19, among of which 34 are co-infected with malaria, and 20 apparently healthy individuals as controls. Only symptomatic COVID-19 patients were included. Patients with other maladies such as diabetes, hypertension and cancers, or those on antioxidant supplements were excluded.

**Study design:** this is a cross sectional analytical study of COVID-19 patients co-infected with malaria in isolation center Dutse, Jigawa Nigeria.

**Study area:** the study was conducted in Dutse metropolis, the Jigawa state capital in the Northwestern Nigeria. At the beginning of this research, Jigawa state not only receives suspected COVID-19 cases within the state, but also from two neighboring state, i.e. Bauchi and Yobe state. Samples collection lasted from March, 2020 to July 2020.

### Collection of specimens (aseptic technique)

*Throat swab:* tonsil and posterior pharyngeal wall was swiped with two swabs simultaneously, and immediately immersed into the tube containing virus transport media (VTM).

*Venous blood:* four milliliters (4mls) of venous blood were collected following aseptic procedure and standard venipuncture without undue pressure to either arm or syringe. A 0.5 mL of the blood was used in making thick blood film and remainder of blood was delivered into a plain container, allowed to clot before centrifuging at 12,000 rpm to obtain sera. The separated sera were stored at -200C until used for analysis of 8-iso PGF2α.

**Statistical analysis:** the statistical analysis was performed using SPSS software (version 26) and Microsoft Excel 16.16.10 for Windows. Specific analyses included Tukey´s HSD (honestly significant difference) test, Brown-Forsythe test, Bartlett's test as well as ANOVA and Student´s -test. The associations between the variables were assessed using Pearson´s correlation coefficient. In all the analyses, the significance in difference was set at p<0.05.

**Diagnosis of COVID-19 using Real Time Polymerase Chain Reaction (PCR):** test principle is based on one-step RT-PCR technique. SARS-COV-2 N and ORF1ab genes were selected as amplification target regions. Specific primers and fluorescent probes were designed for the detection of 2019 Novel Coronavirus RNA in the specimens.

*Nucleic acid extraction and purification:* the extraction of nucleic acids was performed according to manufacturer´s instructions (Life River). Three hundred microliter of the VTM (containing cells from the swabs) was pipetted into centrifuge tube at room temperature. This was followed by inactivation of virus through addition of 20µl proteinase K and 600µl of lysis buffer. The mixture was vortexed and incubated at 56°C for 10 minutes. Six hundred microliter of absolute ethanol was added to the tubes and vortexed for 2 minutes to homogenize. The liquid was carefully transferred to binding column and centrifuged at 12,000rpm for 1 minute. Washing buffers A and W were used in washing at 12,000rpm for 1 minute and membranes were dried immediately to remove residual ethanol by centrifuging at 12,000rpm for 3 minutes. Finally, the nucleic acids were eluted with 50µl of elution buffer after the binding columns were transferred to a new 1.5mL DNase/RNase-free tube. The templates were refrigerated until used for analysis.

*Master mix preparation:* the master mix was prepared according to the manufacturer's (DAAN gene) instruction as follows; 17µl of solution A (ORF1a/N PCR reaction) and 3µl of solution B (ORF1a/N PCR reaction), were added into 1.5ml PCR tube for each sample as follows: Solution A (for 40 sample) = 17 µl x 40 samples = 680µl Solution B (for 40 sample) = 3 µl x 40 samples = 80µl The mixture was vortexed to homogenize for 10 seconds. PCR tubes were arranged facing inwards, 20µl of the master mix were dispensed in each tube including No template control (NTC), positive control (PC), and Negative Control (NC), then 5µl of templates and controls were added accordingly and tightly covered up. The mixture was mixed by inversion and centrifuged for 10 seconds.

**Cycling conditions:** reverse transcription (RT) and holding was done at 45°c for 10 minutes (10: 0) and 95°c for 1 minute thirty seconds (01: 30) and finally amplification was achieved at 95°c for 30 seconds and 58°c for 20 seconds in 45 cycles to detect low copy number targets.

**Figure 1:**
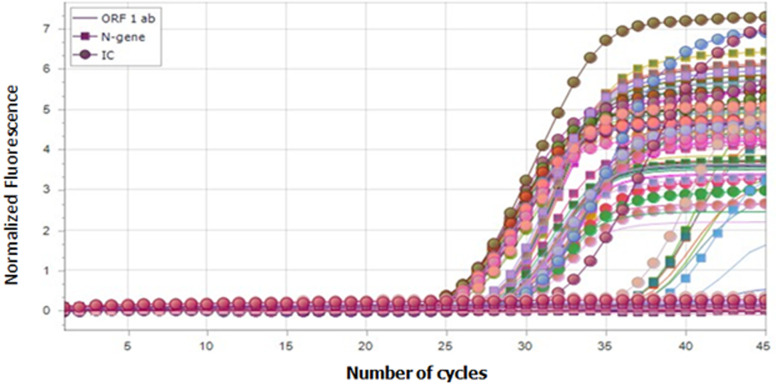
polymerase chain reaction identifier cycling: ORF1ab, N-genes & IC

**Results interpretation:** ≤ 40 threshold cycles (CT) values for internal control, >40 CT or No amplification (N/A) in N-Gene and >40 or N/A in ORF1ab is read as Negative, while ≤ 40 CT for internal control, ≤ 40 for N-Gene and ≤ 40 for ORF1ab is read as Positive. Results of the amplification are shown in (Figure 1).

**Identification of malaria parasite:** microscopy still remains World Health Organization (WHO) recommendation for detection and identification of malaria parasites. Clean grease-free slides were used in preparing a thick blood films, stained with Giemsa; and examined through a microscope with 100X oil immersion objective for the presence of malaria parasites.

*Calculation of Malaria Parasite Density (MPD):* parasites were counted irrespective of whether sexual or asexual forms in relation to a predetermined number of WBCs and an average of 8000/µl (was taken as standard), 200 leucocytes were counted. MPD was calculated using the formula below:

$$\text{Parasites}/\text{μL blood}=\frac{\text{Number of parasites counted}\times \text{8000 white cells}/\text{Ml}}{\text{Number of WBC counted}}$$

**Determination of 8-iso-PGF2α using ELISA technique:** assay principle of 8-iso-PGF2α was based on competitive enzyme-linked immunoassay (ELISA). an antibody to 8-iso-PGF2α was incubated in pre-coated microtiter plate wells. Upon washing, 8-iso-PGF2α standards or treated samples were mixed with an 8-iso PGF2α-HRP conjugate and added simultaneously to the wells. The unconjugated or free 8-iso-PGF2α and 8-iso-PGF2α-HRP conjugate compete for binding to the antibody bound to the plate. After incubation and washing, a substrate to the HRP was added. The HRP activity resulted in color development that is directly proportional to the amount of 8-iso-PGF2α conjugate bound to the plate and inversely proportional to the amount of free 8-iso-PGF2α in the samples or standards.

*Samples preparation:* the samples were treated with 2N NaOH at 45°C for 2 hours in order to hydrolyse lipoproteins and phospholipids for complete measurement of both free and esterified 8-iso-PGF2α. 100ul of 10N HCl per 500 μL of hydrolyzed samples were added, centrifuged for 5 minutes at 12,000 rpm in a microcentrifuge and stored at -200c until used.

*Preparation of standards:* standards were freshly prepared by diluting the 8-iso-PGF2α standard from 200µg/mL to 0.2µg/mL in sample diluents for a 1: 1000 final dilution.

**Colorimetric measurement of alphatocopherol (Vitamin E):** the basic principle is that Fe^3+^can be deoxidized to Fe^2+^ by Vitamin E, Fe^2+^ can react with phenanthroline and form pink compound under certain condition. It´s a two-stage process, firstly, the extraction of n-heptane vitamin E and lastly the addition of chromogenic substances. After colorimetric assay, VE concentration was calculated using formula below:

$$\text{vitamin E (μg/m/L)=}\frac{\text{OD of Sample - OD of Blank }\times \text{C}\times \text{f}\times {\text{2}}^{\text{*}}}{\text{OD of Standard - OD of Blank}}$$

where c=concentration of standard (10µg/m/L); f=Dilution factor of sample before test; 2*= the volume of standard is 0.6 mL, the volume of sample is 0.3 mL, so the sample was condensed twice.

## Results

**Figure 2:**
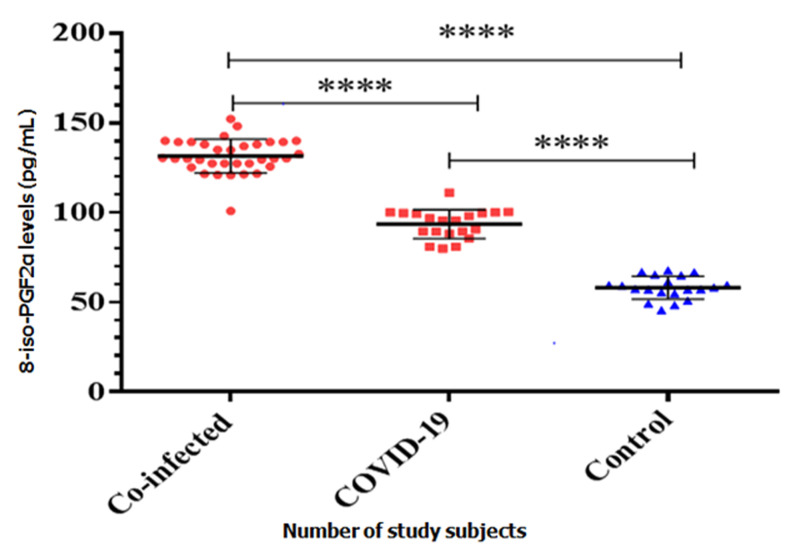
comparison of 8-iso-PGF2α. Scatter plot showing significant increase in 8-iso-PGF2α in both co-infected and COVID-19 groups compared to the control group by one-way ANOVA and Tukey´s multiple comparison post-hoc analysis

**COVID-19 patients co-infected with malaria are predisposed to oxidative stress**: thirty-four (34) COVID-19 patients co-infected with malaria consented to participate in the present research, 22 (64.7%) males and 12 (35.3%) females. The results revealed significant increase of 8-iso-PGF2α and decreased alphatocopherol values among co-infected compared to COVID naïve, p value = .0012 (Figure 2), (Table 1). However the increase in 8-iso-PGF2α levels is not statistically significant between males and females participants, p value = 0.969.

**Table 1:** comparison of 8-iso-PGF2α and Alphatocopherol levels in COVID-19 patient only, co-infected with malaria and control group

Parameters	COVID (n=20)	Co-infected (n=34)	Controls (n=20)	p-value^*^	Level of significance
8-iso-PGF2α (pg/mL)	93.5±8.1	131.6±9.5	58.2±6.3	<0.0001	HS
Alphatocopherol	3.86±0.82	2.40±0.51	9.82±1.09	<0.0001	HS

HS= highly significant; Co-infected=COVID-19 patients co-infected with malaria; n=number of subjects; ^*^determined by one-way ANOVA

**Figure 3:**
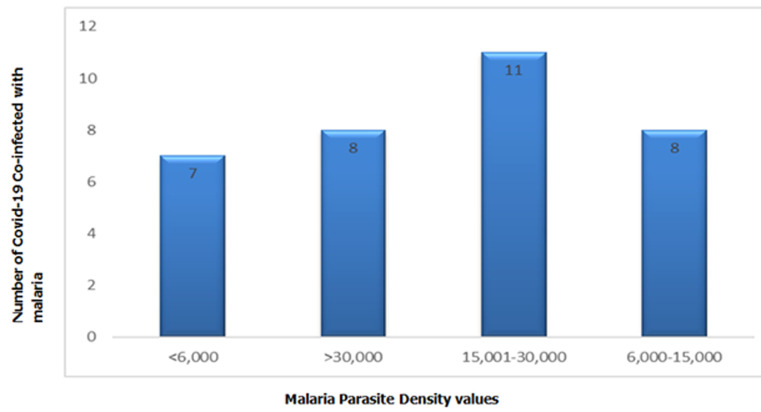
distribution of COVID-19-malaria co-infected patients based on malaria parasite density values

**8-iso-PGF2α level is directly proportional to MPD values among COVID-19 co-infected with malaria:** COVID-19 co-infected patients with malaria were categorically divided into four groups based on MPD values. Of the 34 co-infected subjects 7 (20.6%) have MPD of <6,000, 8(23.5%) have values of 6,000-15,000, while 11(32.4%) and 8(23.5%) have MPD values 16,000-30,000 and >30,000 respectively (Table 2). The results further indicated that 8-iso-PGF2α concentration increases with increase of MPD among COVID-19 co-infected with malaria (Figure 3).

**Table 2:** 8-iso-PGF2α levels in COVID-19 patients co-infected with malaria in relation to malaria parasite density values

MPD values	8-iso-PGF2α (pg/mL)
<6,000	124.8±3.6
6000-15,000	124.4±10.0
16,000-30,000	133.1±5.3
>30,000	142.7±4.9

MPD; Malaria parasite density; 8-iso-PGF2α; 8-isoprostaglandin F2 alpha

**Decreased levels alphatocopherol in COVID-19 co-infected compared to COVID-naïve:** the results were presented as mean ±standard deviation (SD). Serum concentration of vitamin E was determined in the study subjects and it was found that co-infected group have relatively lower levels compared to COVID-19 naïve p value= 0.0011. A Pearson product-moment correlation was conducted to examine the relationship between levels of 8-isoPGF2 alpha and Alphatocopherol among COVID-19 co-infected with malaria. The results revealed strongly negative association = -0.0551, n=34, p < 0.001 (Table 3).

**Table 3:** Pearson correlation test for relationship between serum levels of 8-isoPGF2 and alphatocopherol among COVID-19 co-infected with malaria

	8-isoPGF2alpha	Alphatocopherol
Pearson Correlation	1	-.551^**^
Sig. (2 tailed)		0.001
N	34	34
Pearson Correlation	-.551^**^	
Sig. (2 tailed)	0.001	
N	34	

** Correlation is significant at the 0.001 level (2-tailed); N: number of subjects

## Discussion

The potential impact of COVID-19 on the diagnosis and management of malaria cannot be overemphasized especially in African countries with recognized toll of malaria endemic and other infectious diseases. Current study evaluated the impact on diagnosis and management of COVID-19 co-infected with malaria by exploiting the possible existence of oxidative stress. Results of this present study revealed a significant increase of 8-iso-PGF2α levels among COVID-19 patients co-infected with malaria which may be as a result of accumulated free radicals generated through proinflammatory response such as cytokine storm against SARS-COV-2 infection as well as from Fenton's reaction courtesy of malaria pathogenesis. Generally, double effects of COVID-19 and malaria predisposed the co-infected group more to a high oxidative stress level compared to COVID-19 naïve. Derouiche [11] also suggest that free radicals may be produced as a result of exaggerated immune response orchestrated by SARS-COV-2 infection and NADPH oxidase in the endothelium. Similarly, our findings showed the worst-case scenario involving the co-infected group with higher levels of oxidative stress; as such more antioxidant substances are required to counteract the negative effect of radicalized molecules by terminating or breaking chain reactions. Malaria infection alone induces significant elevation of 8-iso-PGF2α mediated by Fenton’s reaction and additionally co-infection with COVID-19 leads to synergistic 2.3 fold increase of 8-iso-PGF2α levels. This finding is suggestive that 8-iso-PGF2α may be an important predictive marker of cumulative oxidative stress and deterioration of COVID-19 co-infected patient’s condition. Elevation of free radicals and consequent increase of 8-iso-PGF2α may represent a poor prognosis on COVID-19 co-infected patient’s clinical condition. Alphatocopherol being the most important naturally occurring vitamin was assayed among the study groups in order to ascertain its rate of utilization, the findings revealed a significant decrease of the vitamin E in COVID-19 patients compared to control group, however, co-infected persons are more affected with severely decrease vitamin E. concentration. This signifies the protective activity of antioxidant vitamin E towards scavenging free radicals generated as documented by van den Brand *et al*. [12] with regards to SARS-COV infection. Possibility of decreasing the level of free radicals by increasing intracellular antioxidant content through their supplementation could be the key in the management of COVID-19 co-infected with malaria as this may strengthen the immune system, reshuffle oxidant capacities, clear radicals as well as reducing virus’s activity in replication.

Our findings further revealed a negative correlation between 8-iso-PGF2α and Alphatocopherol in COVID-19 subjects. It is therefore recommended to use antioxidant supplements in the management of COVID-19 co-infected with malaria because of demonstrated high free radicals in order to boost their immune system, enhanced antioxidant system and eventually reduces oxidative stress. Finally, current study showed that malaria parasite density is directly proportional to the level of 8-iso-PGF2α and that higher burden of malaria in COVID-19 patients generates too much radicals resulting from overreaction of proinflammatory response due to sequestration of parasites in organs and respiratory cytokine storms. This implies that oxidative stress is notably higher and such patients may have a severer form of the COVID-19. Increased 8-iso-PGF2α in co-infection and decreased alphatocopherol levels can reflect the severity and adverse outcomes compared to COVID-19 naïve because of their tremendous involvement in the pathogenesis and progression of diseases. However, our study has few limitations which includes sample size because of which may not be easy to find relationships between variables and small size may not actually give a representative distribution of a population and this was due to the fact that only 34 patients were found with malaria co-infection during the entire study period. Secondly, Free radicals could have been determined directly but due to their high reactivity, short half-life and unstable nature, it was not possible rather their most reliable and stable metabolite i.e. 8-iso-PGF2α was measured as a marker of oxidative stress. Finally, dearth of relevant and recent information regarding the COVID-19 co-infection with malaria which might have form bases and ultimately be the guide in understanding problem and finding solution to it.

## Conclusion

An inverse correlation of an increase free radicals generation and decrease levels of antioxidant substances has long been documented, current research evaluated serum 8-iso-PGF2α and Alphatocopherol among COVID-19 subjects co-infected with malaria and compared with naïve COVID-19 patients. Conclusively, increase levels of 8-iso-PGF2α was observed among co-infected group compared to COVID-19 naïve, the increase is directly proportional to malaria parasite density and inversely related to Alphatocopherol concentration. It is therefore imperative to include antioxidant therapy for patients with co-infection in order to enhance their clinical outcome.

### What is known about this topic

- It is documented that COVID-19 infections provoked exaggerated proinflammatory response that ultimately leads to ARDS and septic shock;
- Malaria infection is one of the leading cause of death in Nigeria and Sequestration of malaria parasite in vital organs generates oxidative stress.

### What this study adds

- COVID-19 patients co-infected with malaria are associated with elevation of 8-iso-PGF2α levels;
- The increase in 8-iso-PGF2α levels among co-infected is directly proportional to malaria parasite density and inversely related to Alphatocopherol.
